# Effects of perioperative statin use on cardiovascular complications in patients submitted to non-cardiac surgery: protocol for a systematic review, meta-analysis, and trial sequential analysis

**DOI:** 10.1186/s13643-017-0500-0

**Published:** 2017-06-19

**Authors:** Erica Aranha Suzumura, Rodrigo Antonini Ribeiro, Leticia Kawano-Dourado, Pedro Gabriel de Barros e Silva, Claudia Oliveira, Mabel Fernandes Figueiró, Alexandre Biasi Cavalcanti, Renato D. Lopes, Otavio Berwanger

**Affiliations:** 1 Research Institute, Hospital do Coração (HCor), Rua Abílio Soares 250, 12° andar, CEP: 04005-000 São Paulo, SP Brazil; 2 Graduate Program in Epidemiology, Federal University of Rio Grande do Sul, Porto Alegre, Brazil; 3 National Institute of Science and Technology for Health Technology Assessment, CNPq, Brazil; 4 School of Medicine, Faculdade Meridional – IMED, Passo Fundo, Brazil; 5 Pulmonary Division – Heart Institute (InCor) University of Sao Paulo Medical School, São Paulo, Brazil; 6 Duke Clinical Research Institute (DCRI), Durham, NC USA; 7 Brazilian Clinical Research Institute (BCRI), São Paulo, Brazil

**Keywords:** Systematic review, Meta-analysis, Randomized, Statin, Perioperative period, Surgical procedures, Trial sequential analysis

## Abstract

**Background:**

Preliminary evidence suggests statins may reduce major perioperative vascular events. However, evidence is limited to observational studies, underpowered trials, and non-comprehensive systematic reviews. This review aims to assess the effects of perioperative statin use on cardiovascular complications in patients submitted to non-cardiac surgery.

**Methods:**

We will search MEDLINE/PubMed, EMBASE, LILACS, CENTRAL, Web of Science, and CINAHL for randomized controlled trials assessing the effects of perioperative statin use in adults undergoing non-cardiac surgery and reporting cardiovascular complications. For patients already using statins for hyperlipidemia, a preoperative loading dose of statin is required in the experimental group. We will place no language or publication restriction on our search. Teams of two reviewers will independently assess eligibility and risk of bias, and will extract data from the included trials. Our primary outcome is a combination of cardiovascular mortality or non-fatal myocardial infarction. We will also assess the following outcomes: individual components of the primary outcome, all-cause mortality, total myocardial infarction, elevated troponin in the first seven postoperative days, total stroke, total venous thromboembolism, postoperative atrial fibrillation, elevation of creatine phosphokinase or liver enzymes, and rates of myalgia or rhabdomyolysis. We will conduct meta-analyses using random-effects model and will use trial sequential analysis to establish monitoring boundaries to limit global type I error due to repetitive testing for our primary outcome. We will rate the quality of evidence using the GRADE system.

**Discussion:**

The results of this systematic review may help to inform clinical practice and also the design of future large-scale randomized trials.

**Systematic review registration:**

PROSPERO CRD42016035987

**Electronic supplementary material:**

The online version of this article (doi:10.1186/s13643-017-0500-0) contains supplementary material, which is available to authorized users.

## Background

Among the 200 million adults worldwide who undergo non-cardiac surgery annually, over 10 million will suffer a cardiovascular complication within 30 days after surgery [1]. The mechanisms leading to complications such as myocardial infarction are distinct in cardiac and non-cardiac surgeries. Complications related to anastomosis, manipulation of coronaries, myocardial perfusion during extracorporeal circulation, and venous graft thrombosis are associated with cardiac surgeries. Although the mechanisms leading to perioperative myocardial infarction and other vascular complication in non-cardiac surgery are not completely understood, alterations in homeostasis favoring a mismatch of demand and supply of oxygen as well as plaque rupture are thought to play a role [2]. Interventions such as beta-blocker, aspirin, and clonidine have been already assessed in previous randomized controlled trials (RCTs), but none has been shown to be safe and effective on reducing perioperative cardiovascular complication in this setting [3–5].

Statins have a well-established benefit in primary and secondary prevention of cardiovascular events in non-surgical patients [6–8], as well as in patients undergoing cardiac surgery [9]. Their anti-inflammatory and stabilizing effects on atherosclerotic lesions [10–13] make them potential interventions to reduce cardiovascular complications in the perioperative period. Long-term use of statins promotes vasculoprotective effect, mainly mediated by inhibition of the mevalonate pathway and oxidized low density lipoprotein (LDL) generation [14]. They also seem to improve neovascularization in experimental models [15]. Statins also have pleiotropic effects which can be observed even in short-term users. These effects include an anti-inflammatory action, reduction of oxidative stress, stabilization of atheromatous plaques, inhibition of platelet aggregation [16], and effects on endothelial nitric oxide promoting vasorelaxation [17]. Preliminary evidence suggests that even patients already on long-term statin therapy to reduce LDL levels, a loading dose might increase the pleiotropic effects [18]. Small-scale RCTs and observational studies suggest that preoperative statin use may be associated to a large reduction on cardiovascular complications in patients undergoing non-cardiac surgery. However, the evidence is limited by the inherent risk of bias in the observational studies [19] and the low statistical power of the RCTs [20–23].

Previous systematic reviews limited the inclusion of trials to those assessing only statin-naïve patients [22], only trials published in English [22], only trials published as full-texts [23], limited to vascular surgery [21, 23], or conducted search strategy restricted to one database (Medical Literature Analysis and Retrieval System Online (MEDLINE)) [23]. These previous reviews did not calculate the optimal event size for a precise meta-analysis and conducted a trial sequential analysis (TSA) to limit the global type I error [24]. Most of them did not formally assess the quality of the evidence using the Grading of Recommendations Assessment, Development and Evaluation (GRADE) system [25]. Finally, several RCTs have been completed since these reviews were published [26–29], which requires a comprehensive appraisal of current evidence on the use of perioperative statins. Thus, we propose a systematic review to assess the efficacy and safety of perioperative statin use on cardiovascular complications in patients undergoing non-cardiac surgery, including both naïve and patients already on statin therapy. For the latter, statin use is defined as at least a loading dose prior to surgery.

## Methods

Our review protocol has been registered with PROSPERO (registration number CRD42016035987). This protocol is in accordance to the preferred reporting items for systematic review and meta-analysis protocols (PRISMA-P) statement (Additional file 1) [30]. We followed the recommendations of the Cochrane handbook for systematic reviews of interventions for the design and analysis plan development [31], and will follow the preferred reporting items for systematic reviews and meta-analyses (PRISMA) to report the results of this review [32].

### Eligibility

#### Type of study

We will include randomized or quasi-randomized controlled trials with parallel design assessing the efficacy and safety of statins in patients submitted to non-cardiac surgery. Cluster randomized trials will also be included. Randomized cross-over trials will not be included due to carry-over effects of the intervention in the perioperative setting.

#### Type of patients

We will include male and female adult patients undergoing non-cardiac surgery requiring hospital admission. For this review, non-cardiac surgery is defined as a surgical procedure under general or regional anesthesia, not involving manipulation of the heart (pericardium, myocardium, coronaries, and valves). Examples of non-cardiac surgeries that may be included are vascular (aorta aneurysm repair, peripheral vascular bypass, carotid endarterectomy), general (visceral resection, colectomy), thoracic (pneumonectomy, lobectomy), neurosurgery (craniotomy, major spine surgery), orthopedic (hip or knee arthroplasty, internal fixation of bones, arthroscopy), and major urology or gynecology surgeries (nephrectomy, prostatectomy, hysterectomy). Studies involving both cardiac and non-cardiac surgeries will be included in the review; however, only data regarding the subset of patients submitted to non-cardiac surgery will account in the meta-analyses. If not reported in the manuscript, we will e-mail the authors asking for the data. Percutaneous vascular intervention for atherosclerotic obstruction or aneurysm correction will not be considered as non-cardiac surgery for this review.

#### Type of intervention

Perioperative statin use, which characterizes the experimental group for our review, is defined as any use of statin before surgery (including the index day of surgery) that could be continued or not after surgery. For trials including patients already using statins for hypercholesterolemia, perioperative statin use is defined as any loading dose before surgery (including the index day of surgery). Control could be (a) placebo, (b) usual care, or (c) lower dose statin therapy.

### Information sources and search strategy

An experienced librarian and the principal investigator will construct the search strategy. We will search the following electronic databases: MEDLINE/PubMed, Excerpta Medica Database (EMBASE), Latin American and Caribbean Health Sciences Literature (LILACS), Cochrane Central Registry of Controlled Trials (CENTRAL), Web of Science, and the Cumulative Index to Nursing and Allied Health Literature (CINAHL). We will place no language restrictions, and we will use controlled vocabulary whenever possible (MeSH term for MEDLINE and CENTRAL; EMTREE for EMBASE). We will use keywords and their synonyms to sensitize the search and will apply standard filters for the identification of RCTs. We will adapt our MEDLINE search strategy for use with other electronic databases. Additional file 2 shows the planned search strategy.

Additionally, we will hand-search the reference lists of the included studies to identify other relevant trials. Finally, we will attempt to identify unpublished or ongoing trials by contacting experts in the field and by searching clinical trial registries (ClinicalTrials.gov and International Clinical Trials Registry Platform).

### Study selection

Teams of two reviewers (EAS and LKD; RAR and PGMBS) will independently screen all retrieved citations by reviewing titles and abstracts on the computer screen. If at least one of the reviewers considers a citation potentially eligible, the full text will be obtained. If the reviewers cannot be sure whether a study is eligible based on information available on the title and/or abstract, they should retrieve the citation for full text assessment. Then, teams of two reviewers will independently assess the full-text to confirm eligibility and will register reasons for exclusion in a standardized form. We will resolve any disagreement within each team by consensus or third-part adjudication (OB or ABC). Duplicate publications or sub-studies of included trials will be listed under the primary reference, as they may provide additional relevant information that is not available in the original publication.

### Data extraction

Data extraction and risk of bias assessment will also be conducted by two independent reviewers, with any disagreement resolved by consensus or a third part. We will request any further information required from the original authors by e-mail.

We will extract the following data from the included studies: study location, enrollment period, sample size, baseline characteristics of the included patients, details of the experimental and control interventions, length of follow-up, and clinical outcomes. Specifically, we will extract:

- Author
- Year of publication
- Journal of publication
- Type of center participation (single center, Multicenter)
- Study location (institution, city, country)
- Period of study
- Funding sources
- Routine measurement of postoperative cardiac troponins
- If the study included patients already receiving statins
- Type of experimental statin
- Posology of the experimental statin
- Days/hours of administration of statin before the surgery
- Days of administration after the surgery
- Details of the control intervention
- Number of randomized patients in the experimental group and control groups
- Baseline characteristics of the included patients in each group: mean age, number of male patients, number of patients with coronary artery disease, with atrial fibrillation, with dyslipidemia, using statins before the study (chronic users), using beta-blockers, using acetylsalicylic acid or other antiplatelet agent
- Type of non-cardiac surgery performed
- Information to assess risk of bias (as stated in the “Risk of Bias Assessment” section)
- Clinical outcomes (as stated in the “Outcomes” section)

### Risk of bias assessment

Teams of two reviewers will independently assess the risk of bias of the included studies for our primary outcome on a domain-based evaluation. The domains that will be assessed are random sequence generation; allocation concealment; blinding of personnel, patients, and outcome assessors; incomplete outcome data; selective outcome reporting; and early stopping for benefit. We will follow specific instructions of the Cochrane Collaboration’s tool for assessing risk of bias in randomized trials [31]. We will indicate “low risk of bias,” “high risk of bias,” or “unclear” for the domains assessed. We will resolve any disagreement within each team by consensus or third-part adjudication.

### Outcomes

The primary outcome is a combination of cardiovascular mortality or non-fatal myocardial infarction at 30 days. If the authors did not report those outcomes at 30 days, we will consider data at the nearest follow-up period reported. Secondary outcomes are as follows: all-cause mortality; cardiovascular mortality; non-fatal myocardial infarction; total myocardial infarction (fatal and non-fatal myocardial infarction); myocardial injury after noncardiac surgery (MINS) defined as any elevation of cardiac troponins above the cutoff limits as defined by the local laboratory in the first seven postoperative days (as reported in the original studies); total stroke; total venous thromboembolism (deep venous thrombosis or pulmonary embolism); postoperative atrial fibrillation; elevation of creatine phosphokinase (CPK) or liver enzymes; and rates of myalgia or rhabdomyolysis. For eligible studies that did not report any of our outcomes of interest, we will attempt to contact authors by e-mail to obtain unpublished outcome data. We will consider outcome definitions as reported by the individual studies.

### Data synthesis and analysis

We will present the risk ratios (RRs) and their respective 95% confidence intervals (CIs) for the outcomes of each trial. We will perform meta-analyses using the statistical method of Mantel-Haenszel with random-effects model.

#### Subgroup analyses

We will conduct four pre-specified subgroup analyses for the primary outcome:

1. trials categorized as at high overall risk of bias versus trials at low overall risk of bias. We will categorize trials at “low overall risk of bias” those at low risk of bias in all domains assessed, while trials at high risk of bias (or unclear) in at least one domain will be categorize as “high overall risk of bias”.
2. trials that included patients already using statins and received perioperative loading doses versus trials that included only patients that were not under statin therapy.
3. trials grouped according to type of statins. We will create as many subgroups as type of statins included.
4. trials assessing the effect of statins in patients undergoing vascular surgery versus trials involving other surgeries.

#### Meta-regression

We will conduct a meta-regression assessing the effect of time of exposure to statins on our primary outcome. The logarithms of RRs for our primary outcome, weighted by the inverse variance of each study, will be regressed against the number of days or hours before the surgery that statin therapy was initiated.

#### Handling missing data

We will use an intention to treat as primary analysis. To handle patients excluded from the analysis after randomization (missing outcome data), we will conduct a plausible worst-case sensitivity analysis in which all subjects with missing data from one group of the study (the group with the lower event rate) will be assumed to have five times the event rate as those with complete data, and those excluded from the other group were assumed to have the same event rate as subjects with complete data [33, 34]. The rationale for the choice of five times the event rate in subjects lost to follow-up is that in studies examining the rate of events in subjects who are easily followed versus those who are more difficult to follow, five times the event rate was the largest gradient observed [33].

#### Heterogeneity

We will assess statistical heterogeneity across trials or subgroups using Cochrane’s chi-squared test [31] and Higgins’ *I* ^2^ statistics to quantify the percentage of the variability in effect estimates that is due to heterogeneity rather than chance [31, 35]. As Cochrane’s chi-squared test has low power as a comprehensive test of heterogeneity, especially when the number of studies is small, we will consider *P* < 0.10 as statistically significant. We will follow general thresholds to interpret *I* ^2^ results: 0 to 40% may be low heterogeneity; 30 to 60% may represent moderate heterogeneity; 50 to 90% may represent substantial heterogeneity; and 75 to 100% considerable heterogeneity. Nevertheless, final interpretation will consider *I* ^2^ in the context of the specific analysis alongside a qualitative assessment of the combinability of studies [31, 36].

#### Publication bias

We will analyze the probability of publication bias by funnel plot if we identify at least 10 eligible RCTs assessing our primary outcome of interest. We will consider plot asymmetry as suggestive of reporting bias in absence of heterogeneity or evidence of small study effect. We will test for plot asymmetry using Egger’s test [37, 38].

#### Trial sequential analysis

The event size needed for a very precise meta-analysis is at least as large as that for a single optimally powered RCT, so we calculated the optimal event size requirement for our meta-analysis considering a rate of 7.5% of the combined outcome of cardiovascular mortality or non-fatal myocardial infarction in the control group [2–4], relative risk reduction of 20%, 90% of power, and a type I error of 5%. We established the relative risk reduction as 20% to calculate the optimal event size in order to have adequate power to detect even a small but clinically important effect; furthermore, this is the typical effect size observed in perioperative studies [3–5]. Thus, the observation of at least 793 events would be needed. We will conduct a formal TSA [24] by using the optimal event size to help to construct sequential monitoring boundaries for our meta-analysis, analogous to interim monitoring in a single RCT. We will establish the monitoring boundaries limiting the global type I error to 5%. As sensitivity analyses, we will calculate the optimal event size and construct the monitoring boundaries considering a stricter global type I error of 1%, which may be appropriate for meta-analysis of small trials [39].

We will conduct the meta-analyses, subgroup analyses, heterogeneity assessment, and construct funnel plot using the Review Manager Version 5.3 (Cochrane IMS, Oxford, UK). We will conduct meta-regression and Egger’s test using R Software (R Foundation for Statistical Computing, Vienna, Austria). We will use TSA software Version 0.9 Beta (Copenhagen Trial Unit, Copenhagen, Denmark) for trial sequential analysis.

### Quality of evidence

We will classify the quality of evidence generated by this meta-analysis as high, moderate, low, or very low according to the GRADE system [25]. The quality of evidence indicates our certainty of the evidence generated by the meta-analysis of each outcome.

According to the GRADE system, a precise result (associated with low random error or a small *P* value) is necessary but not the only criteria for an evidence to be of high quality. The level of evidence of the meta-analysis will be initially set as high and will be downgraded when any of those were present: study limitations [40], imprecision of estimates of effect [41], indirectness of evidence [42], inconsistency [36], or evidence of reporting bias [43]. An evidence generated by studies with limitations means that most of the included trials showed high risk of bias in at least one domain assessed. “Imprecision” of estimates of effect indicates that there was a non-acceptable random error in the estimate of effect generated by the meta-analysis. “Indirectness” of evidence occurs when there were differences between the population, intervention, comparator, and outcome of interest, and those included in the relevant studies. That is, there is indirectness if the review question was not directly addressed by the available evidence. “Inconsistency” indicates that the results of the individual trials were different from each other. Finally, “reporting bias” occurs when investigators failed to report studies (typically those that show no effect), or outcomes (typically those that may be harmful or for which no effect was observed).

We will use the GRADEpro version 3.6 (GRADE Working Group) to construct the summary of findings table.

## Discussion

Preliminary evidence suggests statins may reduce the incidence of major perioperative cardiovascular complications within 30 days after non-cardiac surgery.

Previous systematic reviews have evaluated the effects of statins in non-cardiac surgery. A Cochrane review pooled the results from three vascular surgery trials (total participants, 178) and found a non-significant decrease in the risk of death and myocardial infarction at 30 days in patients that received statins [21]. The authors considered that the evidence was insufficient to draw conclusions of whether statins resulted in either lower or higher perioperative risks. However, the review was published in 2013 and other trials addressing this research question have been published since then.

A non-registered systematic review [22] assessed the effect of perioperative statins in patients undergoing surgery, including cardiac surgery. Trials involving patients already on statin therapy were excluded. Sixteen RCTs were included. The authors have not stated in the “Methods” section how risk of bias was assessed across the studies. Patients undergoing non-cardiac surgery were evaluated in a subgroup analysis involving only three RCTs. The reviewers showed a decrease in perioperative risk of death (RR = 0.50; 95% CI 0.27–0.91; *P* = 0.02; total of 45 events); however, for this analysis, they considered data of the combined outcome of death from cardiovascular causes or non-fatal myocardial infarction from the study conducted by Schouten et al. (12 combined events in experimental group and 25 in the control group) instead of death events only [44]. Furthermore, subgroup meta-analysis showed a reduction in myocardial infarction rates (RR = 0.53; 95% CI 0.37–0.77; *P* < 0.001; total of 112 events), but pooled number of events from the study conducted by Schouten et al. were also inaccurate. The reviewers included number of patients that showed myocardial ischemia (27 in the experimental group and 47 in the control) instead of myocardial infarction only (8 in the experimental versus 17 in the control) [44].

A more recent systematic review [23] assessed the effects of statins on perioperative outcomes in vascular and endovascular surgery. The authors included four RCTs and 20 observational studies. Search strategy was conducted only in MEDLINE, only studies published as full-texts were considered, and the authors did not obtain unpublished data. Consequently, trials published before the search run date which were eligible for this systematic review were not included [45, 46]. The meta-analysis considering only the RCTs found no difference in all-cause mortality (odds ratio = 0.57; 95% CI 0.27–1.17; *P* = 0.13; total of 30 events) and significant lower incidence of peri-interventional myocardial infarction (odds ratio = 0.44; 95% CI 0.22–0.91; *P* = 0.03; total of 37 events). Due to the low number of events, the analyses were underpowered to provide precise results [47].

Besides, none of the previous systematic reviews calculated the optimal event size and conducted a TSA, nor formally assessed the certainty regarding the gathered evidence using the GRADE system. Hence, a new appraisal of the current evidence on the effect of statin use in perioperative cardiovascular complication is needed, as other trials have been completed since these reviews were published [26–29, 45, 46]. Thus, our systematic review and meta-analysis of RCTs can provide more reliable and powered evidence to better inform clinical practice. If the evidence gathered for our review is not of high quality according to the GRADE system, we then can provide information for the design of an adequately powered and definitive perioperative statin randomized trial.

## Additional files

Additional file 1: PRISMA-P (Preferred Reporting Items for Systematic review and Meta-Analysis Protocols) 2015 checklist: recommended items to address in a systematic review protocol. (DOC 82 kb)

Additional file 2: Planned electronic database search strategy using the following electronic databases: MEDLINE/PubMed, EMBASE, LILACS, CENTRAL, Web of Science, and CINAHL. (DOC 46 kb)

## Abbreviations

CENTRAL: Cochrane Central Registry of Controlled Trials
CI: Confidence interval
CINAHL: Cumulative Index to Nursing and Allied Health Literature
CPK: Creatine phosphokinase
EMBASE: Excerpta Medica Database
GRADE: Grading of Recommendations Assessment, Development and Evaluation
LDL: Low density lipoprotein
LILACS: Latin American and Caribbean Health Sciences Literature
MEDLINE: Medical Literature Analysis and Retrieval System Online
MINS: Myocardial injury after noncardiac surgery
PRISMA: Preferred Reporting Items for Systematic Reviews and Meta-analyses
PRISMA-P: Preferred Reporting Items for Systematic Reviews and Meta-analyses Protocol
PROSPERO: International Prospective Register of Systematic Reviews
RCT: Randomized controlled trial
RR: Risk ratio
TSA: Trial sequential analysis

## References

[1] Weiser TG, Regenbogen SE, Thompson KD, Haynes AB, Lipsitz SR, Berry WR, Gawande AA (2008). An estimation of the global volume of surgery: a modelling strategy based on available data. Lancet.

[2] Lucreziotti S, Carletti F, Santaguida G, Fiorentini C (2006). Myocardial infarction in major noncardiac surgery: epidemiology, pathophysiology and prevention. Heart Int.

[3] Devereaux PJ, Yang H, Yusuf S, Guyatt G, Leslie K, Villar JC, Xavier D, Chrolavicius S, Greenspan L, Pogue J, Pais P, Liu L, Xu S, Málaga G, Avezum A, Chan M, Montori VM, Jacka M, Choi P, POISE Study Group (2008). Effects of extended-release metoprolol succinate in patients undergoing non-cardiac surgery (POISE trial): a randomised controlled trial. Lancet.

[4] Devereaux PJ, Mrkobrada M, Sessler DI, Leslie K, Alonso-Coello P, Kurz A, Villar JC, Sigamani A, Biccard BM, Meyhoff CS, Parlow JL, Guyatt G, Robinson A, Garg AX, Rodseth RN, Botto F, Lurati Buse G, Xavier D, Chan MT, Tiboni M, Cook D, Kumar PA, Forget P, Malaga G, Fleischmann E, Amir M, Eikelboom J, Mizera R, Torres D, Wang CY, VanHelder T, Paniagua P, Berwanger O, Srinathan S, Graham M, Pasin L, Le Manach Y, Gao P, Pogue J, Whitlock R, Lamy A, Kearon C, Baigent C, Chow C, Pettit S, Chrolavicius S, Yusuf S, POISE-2 Investigators (2014). Aspirin in patients undergoing noncardiac surgery. N Engl J Med.

[5] Devereaux PJ, Sessler DI, Leslie K, Kurz A, Mrkobrada M, Alonso-Coello P, Villar JC, Sigamani A, Biccard BM, Meyhoff CS, Parlow JL, Guyatt G, Robinson A, Garg AX, Rodseth RN, Botto F, Lurati Buse G, Xavier D, Chan MT, Tiboni M, Cook D, Kumar PA, Forget P, Malaga G, Fleischmann E, Amir M, Eikelboom J, Mizera R, Torres D, Wang CY, Vanhelder T, Paniagua P, Berwanger O, Srinathan S, Graham M, Pasin L, Le Manach Y, Gao P, Pogue J, Whitlock R, Lamy A, Kearon C, Chow C, Pettit S, Chrolavicius S, Yusuf S, POISE-2 Investigators (2014). Clonidine in patients undergoing noncardiac surgery. N Engl J Med.

[6] Mihaylova B, Emberson J, Blackwell L, Keech A, Simes J, Barnes EH, Voysey M, Gray A, Collins R, Baigent C, Cholesterol Treatment Trialists' (CTT) Collaborators (2012). The effects of lowering LDL cholesterol with statin therapy in people at low risk of vascular disease: meta-analysis of individual data from 27 randomised trials. Lancet.

[7] Fulcher J, O'Connell R, Voysey M, Emberson J, Blackwell L, Mihaylova B, Simes J, Collins R, Kirby A, Colhoun H, Braunwald E, La Rosa J, Pedersen TR, Tonkin A, Davis B, Sleight P, Franzosi MG, Baigent C, Keech A, Cholesterol Treatment Trialists' (CTT) Collaborators (2015). Efficacy and safety of LDL-lowering therapy among men and women: meta-analysis of individual data from 174,000 participants in 27 randomised trials. Lancet.

[8] Baigent C, Blackwell L, Emberson J, Holland LE, Reith C, Bhala N, Peto R, Barnes EH, Keech A, Simes J, Collins R, Cholesterol Treatment Trialists (CTT) Collaboration (2010). Efficacy and safety of more intensive lowering of LDL cholesterol: a meta-analysis of data from 170,000 participants in 26 randomised trials. Lancet.

[9] Hillis LD, Smith PK, Anderson JL, Bittl JA, Bridges CR, Byrne JG (2012). ACCF/AHA guideline for coronary artery bypass graft surgery: executive summary: a report of the American College of Cardiology Foundation/American Heart Association Task Force on Practice Guidelines. J Thorac Cardiovasc Surg..

[10] Li J, Li JJ, He JG, Nan JL, Guo YL, Xiong CM (2010). Atorvastatin decreases C-reactive protein-induced inflammatory response in pulmonary artery smooth muscle cells by inhibiting nuclear factor-kappaB pathway. Cardiovasc Ther.

[11] Liakopoulos OJ, Dorge H, Schmitto JD, Nagorsnik U, Grabedunkel J, Schoendube FA (2006). Effects of preoperative statin therapy on cytokines after cardiac surgery. Thorac Cardiovasc Surg.

[12] Mitani H, Egashira K, Kimura M (2003). HMG-CoA reductase inhibitor, fluvastatin, has cholesterol-lowering independent “direct” effects on atherosclerotic vessels in high cholesterol diet-fed rabbits. Pharmacol Res.

[13] Patti G, Pasceri V, Colonna G, Miglionico M, Fischetti D, Sardella G, Montinaro A, Di Sciascio G (2007). Atorvastatin pretreatment improves outcomes in patients with acute coronary syndromes undergoing early percutaneous coronary intervention: results of the ARMYDA-ACS randomized trial. J Am Coll Cardiol.

[14] Fang SY, Roan JN, Luo CY, Tsai YC, Lam CF (2013). Pleiotropic vascular protective effects of statins in perioperative medicine. Acta Anaesthesiol Taiwan..

[15] Chiang KH, Cheng WL, Shih CM, Lin YW, Tsao NW, Kao YT (2015). Statins, HMG-CoA reductase inhibitors, improve neovascularization by increasing the expression density of CXCR4 in endothelial progenitor cells. PLoS One..

[16] Wang CY, Liu PY, Liao JK (2008). Pleiotropic effects of statin therapy: molecular mechanisms and clinical results. Trends Mol Med.

[17] Bates K, Ruggeroli CE, Goldman S, Gaballa MA (2002). Simvastatin restores endothelial NO-mediated vasorelaxation in large arteries after myocardial infarction. Am J Physiol Heart Circ Physiol..

[18] Cay S, Cagirci G, Sen N, Balbay Y, Durmaz T, Aydogdu S (2010). Prevention of peri-procedural myocardial injury using a single high loading dose of rosuvastatin. Cardiovasc Drugs Ther.

[19] Berwanger O, Le Manach Y, Suzumura EA, Biccard B, Srinathan SK, Szczeklik W, Santo JA, Santucci E, Cavalcanti AB, Archbold RA, Devereaux PJ (2016). VISION Investigators. Association between pre-operative statin use and major cardiovascular complications among patients undergoing non-cardiac surgery: the VISION study. Eur Heart J.

[20] Kapoor AS, Kanji H, Buckingham J, Devereaux PJ, McAlister FA (2006). Strength of evidence for perioperative use of statins to reduce cardiovascular risk: systematic review of controlled studies. BMJ.

[21] Sanders RD, Nicholson A, Lewis SR, Smith AF, Alderson P (2013). Perioperative statin therapy for improving outcomes during and after noncardiac vascular surgery. The Cochrane database of systematic reviews..

[22] de Waal BA, Buise MP, van Zundert AA (2015). Perioperative statin therapy in patients at high risk for cardiovascular morbidity undergoing surgery: a review. Br J Anaesth.

[23] Antoniou GA, Hajibandeh S, Hajibandeh S, Vallabhaneni SR, Brennan JA, Torella F (2015). Meta-analysis of the effects of statins on perioperative outcomes in vascular and endovascular surgery. J Vasc Surg.

[24] Pogue J, Yusuf S (1997). Cumulating evidence from randomized trials: utilizing sequential monitoring boundaries for cumulative meta-analysis. Control Clin Trials.

[25] Guyatt GH, Oxman AD, Schünemann HJ (2011). GRADE guidelines: a new series of articles in the Journal of Clinical Epidemiology. J Clin Epidemiol.

[26] Shyamsundar M, McAuley DF, Shields MO, MacSweeney R, Duffy MJ, Johnston JR, McGuigan J, Backman JT, Calfee CS, Matthay MM, Griffiths MJ, McDowell C, Elborn SJ, OʼKane CM (2014). Effect of simvastatin on physiological and biological outcomes in patients undergoing esophagectomy: a randomized placebo-controlled trial. Ann Surg.

[27] Xia J, Qu Y, Shen H, Liu X (2014). Patients with stable coronary artery disease receiving chronic statin treatment who are undergoing noncardiac emergency surgery benefit from acute atorvastatin reload. Cardiology.

[28] Xia J, Qu Y, Yin C, Xu D (2015). Preoperative rosuvastatin protects patients with coronary artery disease undergoing noncardiac surgery. Cardiology.

[29] Berwanger O, de Barros E Silva PG, Barbosa RR, Precoma DB, Figueiredo EL, Hajjar LA, Kruel CD, Alboim C, Almeida AP, Dracoulakis MD, Filho HV, Carmona MJ, Maia LN, De Oliveira Filho JB, Saraiva JF, Soares RM, Damiani L, Paisani D, Kodama AA, Gonzales B, Ikeoka DT, Devereaux PJ, Lopes RD, LOAD Investigators (2017). Atorvastatin for high-risk statin-naïve patients undergoing noncardiac surgery: The Lowering the Risk of Operative Complications Using Atorvastatin Loading Dose (LOAD) randomized trial. Am Heart J.

[30] Moher D, Shamseer L, Clarke M, Ghersi D, Liberati A, Petticrew M, Shekelle P, Stewart LA, PRISMA-P Group (2015). Preferred reporting items for systematic review and meta-analysis protocols (PRISMA-P) 2015 statement. Syst Rev.

[31] Higgins JPT, Green S (editors). Cochrane Handbook for Systematic Reviews of Interventions Version 5.1.0 [updated March 2011]. The Cochrane Collaboration, 2011. Available from www.handbook.cochrane.org. Accessed 10 May 2017.

[32] Moher D, Liberati A, Tetzlaff J, Altman DG (2009). PRISMA Group. Preferred reporting items for systematic reviews and meta-analyses: the PRISMA statement. J Clin Epidemiol.

[33] Akl EA, Kahale LA, Agoritsas T, Brignardello-Petersen R, Busse JW, Carrasco-Labra A, Ebrahim S, Johnston BC, Neumann I, Sola I, Sun X, Vandvik P, Zhang Y, Alonso-Coello P, Guyatt G (2015). Handling trial participants with missing outcome data when conducting a meta-analysis: a systematic survey of proposed approaches. Syst Rev..

[34] Akl EA, Johnston BC, Alonso-Coello P, Neumann I, Ebrahim S, Briel M, Cook DJ, Guyatt GH (2013). Addressing dichotomous data for participants excluded from trial analysis: a guide for systematic reviewers. PLoS One.

[35] Higgins JP, Thompson SG, Deeks JJ, Altman DG (2003). Measuring inconsistency in meta-analyses. BMJ.

[36] Guyatt GH, Oxman AD, Kunz R, Woodcock J, Brozek J, Helfand M, Alonso-Coello P, Glasziou P, Jaeschke R, Akl EA, Norris S, Vist G, Dahm P, Shukla VK, Higgins J, Falck-Ytter Y, Schünemann HJ, GRADE Working Group (2011). GRADE guidelines: 7. Rating the quality of evidence—inconsistency. J Clin Epidemiol.

[37] Sterne JA, Egger M, Smith GD (2001). Systematic reviews in health care: Investigating and dealing with publication and other biases in meta-analysis. BMJ.

[38] Egger M, Smith GD (1998). Bias in location and selection of studies. BMJ.

[39] Devereaux PJ, Beattie WS, Choi PT, Badner NH, Guyatt GH, Villar JC, Cinà CS, Leslie K, Jacka MJ, Montori VM, Bhandari M, Avezum A, Cavalcanti AB, Giles JW, Schricker T, Yang H, Jakobsen CJ, Yusuf S (2005). How strong is the evidence for the use of perioperative beta blockers in non-cardiac surgery? Systematic review and meta-analysis of randomised controlled trials. BMJ.

[40] Guyatt GH, Oxman AD, Vist G, Kunz R, Brozek J, Alonso-Coello P, Montori V, Akl EA, Djulbegovic B, Falck-Ytter Y, Norris SL, Williams JW, Atkins D, Meerpohl J, Schünemann HJ (2011). GRADE guidelines: 4. Rating the quality of evidence—study limitations (risk of bias). J Clin Epidemiol.

[41] Guyatt GH, Oxman AD, Kunz R, Brozek J, Alonso-Coello P, Rind D, Devereaux PJ, Montori VM, Freyschuss B, Vist G, Jaeschke R, Williams JW, Murad MH, Sinclair D, Falck-Ytter Y, Meerpohl J, Whittington C, Thorlund K, Andrews J, Schünemann HJ (2011). GRADE guidelines 6. Rating the quality of evidence—imprecision. J Clin Epidemiol.

[42] Guyatt GH, Oxman AD, Kunz R, Woodcock J, Brozek J, Helfand M, Alonso-Coello P, Falck-Ytter Y, Jaeschke R, Vist G, Akl EA, Post PN, Norris S, Meerpohl J, Shukla VK, Nasser M, Schünemann HJ, GRADE Working Group (2011). GRADE guidelines: 8. Rating the quality of evidence—indirectness. J Clin Epidemiol.

[43] Guyatt GH, Oxman AD, Montori V, Vist G, Kunz R, Brozek J, Alonso-Coello P, Djulbegovic B, Atkins D, Falck-Ytter Y, Williams JW, Meerpohl J, Norris SL, Akl EA, Schünemann HJ (2011). GRADE guidelines: 5. Rating the quality of evidence—publication bias. J Clin Epidemiol.

[44] Schouten O, Boersma E, Hoeks SE, Benner R, van Urk H, van Sambeek MR, Verhagen HJ, Khan NA, Dunkelgrun M, Bax JJ, Poldermans D (2009). Dutch echocardiographic cardiac risk evaluation applying stress echocardiography study group. Fluvastatin and perioperative events in patients undergoing vascular surgery. N Engl J Med.

[45] Medvedeva EA, Shchukin Iu V, Seleznev EI. Prevention of cardiovascular complications in patients undergoing aortic-iliac reconstructions by correction of inflammation and endotoxemia. Vestn Ross Akad Med Nauk. 2013;(2):24–8. 10.15690/vramn.v68i2.545 23819325

[46] Almeida R, Coelho OR, Meneses FH, Oliveira G, Molinari G, Franca R (2010). The APVS (atorvastatin in the perioperative of vascular surgery) study. Abstract from World Congress of Cardiology Scientific Sessions 2010, Beijing China. Circulation.

[47] Yusuf S, Peto R, Lewis J, Collins R, Sleight P (1985). Beta blockade during and after myocardial infarction: an overview of the randomized trials. Prog Cardiovasc Dis.

