# Supplementary material for: Effects of perioperative statin use on cardiovascular complications in patients submitted to non-cardiac surgery: protocol for a systematic review, meta-analysis, and trial sequential analysis
Source: Syst Rev. 2017 Jun 19;6:116. doi: 10.1186/s13643-017-0500-0 (PMC5477118; doi:10.1186/s13643-017-0500-0)
Supplement: Supplementary file 2 — Planned electronic database search strategy using the following electronic databases: MEDLINE/PubMed, EMBASE, LILACS, CENTRAL, Web of Science, and CINAHL. (DOC 46 kb) [file 13643_2017_500_MOESM2_ESM.doc]

**Additional file 2: Planned electronic database search strategy**

| **Database** | **Search strategy** |
| --- | --- |
| MEDLINE/  PubMed | ("Hydroxymethylglutaryl-CoA Reductase Inhibitors"[Mesh] OR statin OR statins OR simvastatin OR rosuvastatin OR fluvastatin OR cerivastatin OR lovastatin OR pravastatin OR atorvastatin) AND ("General Surgery"[Mesh] OR surgery OR surgery[MeSH Subheading] OR "Surgical Procedures, Operative"[Mesh] OR Operative Surgical Procedure OR Operative Procedures OR noncardiac surgery OR non-cardiac surgery OR "Noncardiovascular Surgery" OR "Intraoperative Period"[Mesh] OR Intraoperative Periods OR Period, Intraoperative OR Periods, Intraoperative OR Intraoperative OR "Postoperative Period"[Mesh] OR Period, Postoperative OR Periods, Postoperative OR Postoperative Periods OR Postoperative OR "Perioperative Period"[Mesh] OR Period, Perioperative OR Periods, Perioperative OR Perioperative Periods OR Perioperative OR "Preoperative Period"[Mesh] OR Period, Preoperative OR preoperative OR "Postoperative Care"[Mesh] OR Care, Postoperative OR Postoperative Procedures OR Postoperative Procedure OR Procedure, Postoperative OR Procedures, Postoperative OR Postoperative care OR "Intraoperative Care"[Mesh] OR care, perioperative OR perioperative care OR care, intraoperative OR intraoperative care OR "Preoperative Care"[Mesh] OR Care, Preoperative OR Preoperative Procedure OR Procedure, Preoperative OR Procedures, Preoperative OR Preoperative Procedures OR preoperative care OR "Recovery Room"[Mesh] OR Recovery Rooms, Hospital OR Room, Hospital Recovery OR Rooms, Hospital Recovery OR Hospital Recovery Rooms OR Recovery Rooms OR Room, Recovery OR Rooms, Recovery OR Recovery Room, Hospital OR Hospital Recovery Room) AND (randomized controlled trial [pt] OR controlled clinical trial [pt] OR randomized [tiab] OR placebo [tiab] OR drug therapy [sh] OR randomly [tiab] OR trial [tiab] OR groups [tiab] NOT (animals[mh] NOT humans[mh])) |
| Cochrane Library / CENTRAL | MeSH:[Hydroxymethylglutaryl-CoA Reductase Inhibitors]/ exp OR (statin OR statins OR simvastatin OR rosuvastatin OR fluvastatin OR cerivastatin OR lovastatin OR pravastatin OR atorvastatin):ti,ab,kw AND MeSH:[General Surgery] /exp OR surgery:ti,ab,kw OR MeSH:[Surgical Procedures, Operative] /exp OR (Operative Surgical Procedure OR Operative Procedures OR noncardiac surgery OR non-cardiac surgery OR "Noncardiovascular Surgery"):ti,ab,kw OR MeSH:[Intraoperative Period]/ exp OR (Intraoperative Periods OR Period, Intraoperative OR Periods, Intraoperative OR Intraoperative):ti,ab,kw OR MeSH:[Intraoperative Period]/ exp OR (Period, Postoperative OR Periods, Postoperative OR Postoperative Periods OR Postoperative):ti,ab,kw OR MeSH:[Perioperative Period]/ exp OR (Period, Perioperative OR Periods, Perioperative OR Perioperative Periods OR Perioperative):ti,ab,kw OR MeSH:[Preoperative Period]/ exp OR (Period, Preoperative OR preoperative):ti,ab,kw OR MeSH:[Postoperative Care]/exp OR (Care, Postoperative OR Postoperative Procedures OR Postoperative Procedure OR Procedure, Postoperative OR Procedures, Postoperative OR Postoperative care):ti,ab,kw OR MeSH:[Intraoperative Care]/ exp OR (care, perioperative OR perioperative care OR care, intraoperative OR intraoperative care):ti,ab,kw OR MeSH:[Preoperative Care]/ exp OR (Care, Preoperative OR Preoperative Procedure OR Procedure, Preoperative OR Procedures, Preoperative OR Preoperative Procedures OR preoperative):ti,ab,kw OR MeSH:[Recovery Room]/ exp OR (Recovery Rooms, Hospital OR Room, Hospital Recovery OR Rooms, Hospital Recovery OR Hospital Recovery Rooms OR Recovery Rooms OR Room, Recovery OR Rooms, Recovery OR Recovery Room, Hospital OR Hospital Recovery Room):ti,ab,kw |
| EMBASE | 'hydroxymethylglutaryl coenzyme a reductase inhibitor'/exp OR statin OR statins OR simvastatin OR rosuvastatin OR fluvastatin OR cerivastatin OR lovastatin OR  pravastatin OR atorvastatin AND 'general surgery'/mj OR 'surgery'/mj OR 'operative  surgical procedure' OR 'operative procedures' OR 'noncardiac surgery' OR 'non-cardiac surgery' OR 'noncardiovascular surgery' OR 'intraoperative period'/mj OR 'intraoperative periods' OR 'period, intraoperative' OR 'periods, intraoperative' OR   intraoperative OR 'postoperative period'/mj OR 'period, postoperative' OR 'periods, postoperative' OR 'postoperative periods' OR postoperative OR 'perioperative period'/mj OR 'period, perioperative' OR 'periods, perioperative' OR 'perioperative periods' OR perioperative OR 'preoperative period'/mj OR 'period, preoperative' OR  preoperative OR 'postoperative care'/mj OR 'care, postoperative' OR 'postoperative  procedures' OR 'postoperative procedure' OR 'procedure, postoperative' OR  'procedures, postoperative' OR 'postoperative care' OR 'peroperative care'/mj OR 'care, perioperative' OR 'perioperative care' OR 'care, intraoperative' OR  'intraoperative care' OR 'preoperative care'/mj OR 'care, preoperative' OR  'preoperative procedure' OR 'procedure, preoperative' OR 'procedures, preoperative' OR 'preoperative procedures' OR 'preoperative care'  OR 'recovery room'/mj OR 'recovery rooms, hospital' OR 'room, hospital recovery' OR 'rooms, hospital recovery' OR 'hospital recovery rooms’ OR 'recovery rooms' OR 'room,  recovery' OR 'rooms, recovery' OR 'recovery room, hospital' OR 'hospital recovery room' AND [embase]/lim AND 'randomized controlled trial'/exp OR 'randomized controlled trial' OR 'controlled clinical trial'/exp OR 'controlled clinical trial' OR 'randomization'/exp OR 'randomization' OR 'double blind procedure'/exp OR 'double blind procedure' OR 'single blind procedure'/exp OR 'single blind procedure' OR  'clinical trial'/exp OR 'clinical trial'/syn OR  'clinical trial' OR (singl* OR doubl* OR  trebl* OR tripl* AND (mask* OR 'blind'/syn OR 'blind')) OR  'placebo'/exp OR 'placebo' OR placebo* OR random* OR 'crossover procedure'/syn OR 'crossover procedure' NOT ('animal'/exp OR 'animal' NOT ('human'/exp OR 'human')) AND [embase]/lim |
| LILACS | (Inibidores de Hidroximetilglutaril-CoA Redutases OR Inhibidores de Hidroximetilglutaril-CoA Reductasas OR statin OR statins OR simvastatin OR rosuvastatin OR fluvastatin OR cerivastatin OR lovastatin OR pravastatin OR atorvastatin) AND (Cirurgia Geral OR General Surgery OR Cirugía General OR cirurgia OR surgery OR cirugía OR non-cardiac surgery) |
| Web of Science | Tópico:(Hydroxymethylglutaryl-CoA Reductase Inhibitors OR statin OR statins OR simvastatin OR rosuvastatin OR fluvastatin OR cerivastatin OR lovastatin OR  pravastatin OR atorvastatin) AND Tópico:(General Surgery OR surgery OR Surgical Procedures Operative OR  Operative Surgical Procedure OR Operative Procedures) OR noncardiac surgery OR non-cardiac surgery OR Noncardiovascular Surgery OR Intraoperative Period OR Intraoperative Periods  OR Period Intraoperative OR Periods Intraoperative OR Intraoperative OR Postoperative Period OR Period Postoperative OR Periods Postoperative OR  Postoperative Periods OR Postoperative OR Perioperative Period OR Period  Perioperative OR Periods Perioperative OR Perioperative Periods OR Perioperative  OR Preoperative Period OR Period Preoperative OR Preoperative OR Postoperative Care OR Care Postoperative OR Postoperative Procedures OR  Postoperative Procedure OR Procedure Postoperative OR Procedures Postoperative OR Postoperative care OR Care Postoperative OR Postoperative Procedures OR Postoperative Procedure OR Procedure Postoperative OR  Procedures Postoperative OR Postoperative care OR Intraoperative Care OR care perioperative OR perioperative care OR care intraoperative OR intraoperative care OR Preoperative Care OR Care Preoperative OR Preoperative Procedure OR Procedure Preoperative OR Procedures Preoperative OR Preoperative Procedures  OR Preoperative care OR Recovery Rooms Hospital OR Rooms Hospital Recovery  OR Hospital Recovery Rooms OR Recovery Rooms OR Room Recovery OR Rooms Recovery OR  Recovery Room Hospital OR Hospital Recovery Room) AND Tópico:(randomized controlled trial* OR controlled clinical trial OR random allocation OR double-blind method OR single-blind method OR clinical trial* OR placebo* OR comparative study OR follow-up studies evaluation studies) |
| CINAHL | (TX Hydroxymethylglutaryl-CoA Reductase Inhibitors OR statin OR statins OR simvastatin OR rosuvastatin OR fluvastatin OR cerivastatin OR lovastatin OR pravastatin OR atorvastatin) AND (TX General Surgery OR surgery OR Surgical Procedures, Operative OR Operative Surgical Procedure OR Operative Procedures OR noncardiac surgery OR non-cardiac surgery OR Noncardiovascular Surgery OR Intraoperative Period OR Intraoperative Periods OR Period, Intraoperative OR Periods, Intraoperative OR Intraoperative OR Postoperative Period OR Period, Postoperative OR Periods, Postoperative OR Postoperative Periods OR Postoperative OR Perioperative Period OR Period, Perioperative OR Periods, Perioperative OR Perioperative Periods OR Perioperative OR Preoperative Period OR Period, Preoperative OR preoperative OR Postoperative Care OR Care, Postoperative OR Postoperative Procedures OR Postoperative Procedure OR Procedure, Postoperative OR Procedures, Postoperative OR Postoperative care OR Intraoperative Care OR care, perioperative OR perioperative care OR care, intraoperative OR intraoperative care OR Preoperative Care OR Care, Preoperative OR Preoperative Procedure OR Procedure, Preoperative OR Procedures, Preoperative OR Preoperative Procedures OR preoperative care) AND (TX randomized controlled trials OR rct OR randomised control trials) |
